# Adaptive multimode signal reconstruction from time–frequency representations

**DOI:** 10.1098/rsta.2015.0205

**Published:** 2016-04-13

**Authors:** Sylvain Meignen, Thomas Oberlin, Philippe Depalle, Patrick Flandrin, Stephen McLaughlin

**Affiliations:** 1 Laboratoire Jean Kuntzmann (UMR CNRS 5224), Université Joseph Fourier, 51 rue des Mathématiques, Campus de Saint Martin d’Hères, BP 53, 38041 Grenoble Cedex 09, France; 2 IRIT and INP-ENSEEIHT (UMR CNRS 5505), Université de Toulouse, 2 rue Charles Camichel, BP 7122, 31071 Toulouse Cedex 07, France; 3 Centre for Interdisciplinary Research in Music Media and Technology (CIRMMT), McGill University, 527 Sherbrooke Street West, Montreal, Quebec, Canada H3A 1E3; 4 Laboratoire de Physique (UMR CNRS 5672), ENS de Lyon, Université de Lyon, 46 allée d’Italie, 69364 Lyon Cedex 07, France; 5 School of Engineering and Physical Sciences, Heriot-Watt University, Riccarton, Edinburgh EH14 4AS, UK

**Keywords:** time–frequency, AM–FM signals, reassignment, multimode signal reconstruction

## Abstract

This paper discusses methods for the adaptive reconstruction of the modes of multicomponent AM–FM signals by their time–frequency (TF) representation derived from their short-time Fourier transform (STFT). The STFT of an AM–FM component or mode spreads the information relative to that mode in the TF plane around curves commonly called *ridges*. An alternative view is to consider a mode as a particular TF domain termed a *basin of attraction*. Here we discuss two new approaches to mode reconstruction. The first determines the ridge associated with a mode by considering the location where the direction of the reassignment vector sharply changes, the technique used to determine the basin of attraction being directly derived from that used for ridge extraction. A second uses the fact that the STFT of a signal is fully characterized by its zeros (and then the particular distribution of these zeros for Gaussian noise) to deduce an algorithm to compute the mode domains. For both techniques, mode reconstruction is then carried out by simply integrating the information inside these basins of attraction or domains.

## 1. Introduction

In the signal processing community, the meaning of frequency is well understood, and over two centuries, we have developed a series of mathematical tools to enable us to analyse signals in terms of the energy distribution in frequency. However, as with many obvious concepts, when we deal with signals that contain multiple components and these components are time-varying, life becomes more difficult. The time–frequency (TF) analysis tools we have are often not appropriate or the results are problematic to interpret. In this paper, we focus on the adaptive reconstruction of the modes of a multicomponent signal, consisting of AM–FM modes, by using the TF representation derived from the short-time Fourier transform (STFT) of the signal.

The past 40 years have seen numerous TF methods proposed (e.g. [1–3] for surveys). In the methods for the analysis of AM–FM signals, the idealized scenario is that these signals correspond to a perfectly localized trajectory associated with the instantaneous frequency in the TF plane. Kodera, Gendrin & de Villedary pioneered an approach that modified the STFT [4]. They pointed out that spreading the STFT magnitude can be compensated for by taking into account the phase information usually discarded. Subsequently, there was development of Wigner-type distributions, tailored to guarantee localization of signals with specific FM laws, though at the expense of cross-terms that were problematic in the multicomponent case. Auger & Flandrin (who coined the term *reassignment*) showed that the explicit use of the STFT phase can be efficiently replaced by a combination of STFTs with suitable windows [5]. Maes & Daubechies then developed *synchrosqueezing* [6], a special case of reassignment, with the additional advantage of enabling reconstruction. Such phase-based, data-driven methods have recently gained a renewed interest (e.g. the review paper [7]), and it is from here that the methods described in this paper begin.

The STFT of an AM–FM component or mode spreads the information relative to that mode in the TF plane around a curve commonly called a *ridge*. Conventionally, the focus of signal reconstruction has been on dealing directly with these ridges. In this paper, we develop an alternative view by considering a mode as a particular TF domain which we term a *basin of attraction* (an early attempt in such a direction can be found in [8]). In this paper, we focus on two approaches. The first determines the ridge associated with a mode by considering either the local maxima of the spectrogram in some predefined direction [9] or the zeros of the ridge points, in relation to *reassignment* techniques [5,10]; the technique used to determine the basin of attraction is derived directly from the method for ridge extraction. The second exploits the fact that the STFT of a signal is fully characterized by its zeros [11] and then exploits the distribution of these zeros for Gaussian noise to deduce an algorithm that computes the mode domains. Mode reconstruction is then carried out by simply integrating the information inside these domains. Because the zeros of the STFT and the maxima of its modulus can both be used for mode reconstruction, the goal of this paper is to draw parallels and to highlight the differences between these two approaches and show their relevance in noisy situations and where the number of modes varies with time. One final remark to note is that the proposed approaches are expected to be beneficial for synchrosqueezing methods (e.g. [12] for recent advances), because determining basins is a prerequisite for reconstruction.

The paper is structured as follows. First of all, we introduce the basic analysis tools used for TF analysis (§2), such as the STFT and reassignment coupled with a discussion of the zeros of the STFT. Then, the two different techniques which are the core of this paper and which compute the basins of attraction and mode domains are presented and discussed (§3). We then present some numerical experiments (§4) to validate these methods when applied to a variety of synthetic and natural signals. Finally, we draw some conclusions (§5) where we seek to highlight open issues worthy of further exploration.

## 2. Tools for time–frequency analysis

Here, we briefly review the basics associated with TF analysis that are relevant to this paper; specifically, the STFT, ridges and their relationship to the modes of multicomponent signals and finally a brief description of the principles of reassignment. We then proceed to briefly discuss the significance of the zeros of the STFT, and how they may be used.

### (a) The short-time Fourier transform

Given a signal 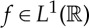, the space of real integrable functions, we define its Fourier transform by
2.1
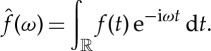
The *short-ime Fourier transform* (STFT) of signal *f* is then defined by
2.2
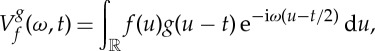
where *g* is assumed to be a real-valued window with *L*^2^ norm equal to 1. The spectrogram is then defined as 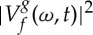. In the following, we will make extensive use of the unit-energy Gaussian window defined as
2.3
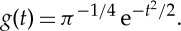


### (b) Ridges and multicomponent signals

A multicomponent signal is a superposition of AM/FM waves of the form
2.4
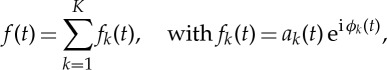
for some finite *K*, where *a*_*k*_(*t*)>0 is a continuously differentiable function, *ϕ*_*k*_ is a two times continuously differentiable function satisfying *ϕ*_*k*_′(*t*)>0 and *ϕ*_*k*+1_′(*t*)>*ϕ*_*k*_′(*t*) for all *t*.

The general form for the STFT of a multicomponent signal admits the following first-order approximation assuming *a*_*k*_′(*t*)≤*ε* and *ϕ*_*k*_′′(*t*)≤*ε*′[13,14]:
2.5
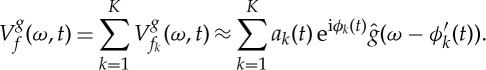
We can further assume that the modes satisfy the following separation condition:
2.6
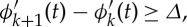
where Δ is called the *separation parameter*. In this case, using equation (2.5) and assuming the essential frequency support of the window *g* is [−Δ,Δ], the components occupy distinct domains of the TF plane, allowing for their separation. In the following, *f*_*k*_ will be referred to as an AM–FM component. Going further 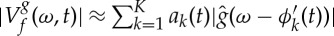, so that each mode *f*_*k*_ is associated with a TF *ridge* corresponding roughly to the (*t*,*ϕ*_*k*_′(*t*)) curve provided 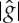 attains its maximum at 0. The detection of ridges and their use in mode reconstruction has been pioneered in [13] and subsequently developed in a number of works (e.g. [14] or [15]).

### (c) Reassignment basics

Here, the principle underlying the *reassignment method* (RM) in the STFT context, which we will use later for ridge extraction, is introduced. The aim of RM is to compensate for the TF shifts induced by the two-dimensional smoothing which defines the STFT. To do so, a meaningful TF location to which the local energy given by the spectrogram is assigned, is first determined [5]. This corresponds to the centroid of the distribution, whose coordinates are defined by
2.7
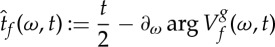
and
2.8
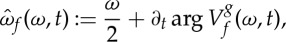
at any point (*ω*,*t*) such that 
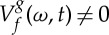
. Both quantities, which *locally* define an instantaneous frequency and a group delay, enable perfect localization of linear chirps [5]. An efficient procedure computes them according to
2.9
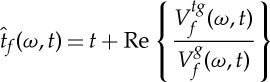
and
2.10
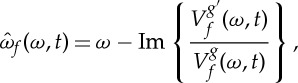
where *tg* stands for the function *tg*(*t*) and Re{*Z*} (resp. Im{*Z*}) is the real (resp. imaginary) part of the complex number *Z*. The RM is naturally associated with a so-called *reassignment vector* (RV) defined by
2.11
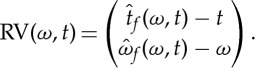
Assuming the window *g* is the Gaussian window with unit variance defined in (2.3), then 
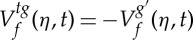
, and thus RV can be rewritten as
2.12
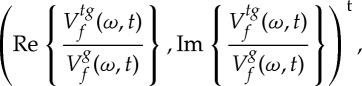
which can, in turn, be expressed in terms of the modulus of the STFT as follows [16]:
2.13
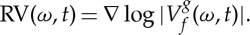
This last expression suggests the relationship between RV and local extrema of the amplitude of the STFT: RV(*ω*,*t*) is the null vector if and only if 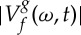 admits a local extremum along both time and frequency directions. Moreover, (2.13) indicates that RVs tend to point towards local maxima that can be interpreted as attractors, whereas zeros act as repellers: this will be discussed and used in the following.

### (d) On the zeros of the short-time Fourier transform

Rather than considering time and frequency independently, it can be interesting to consider them as the real and imaginary parts of a complex-valued variable, thus identifying the TF plane with the complex plane. Doing so by the introduction of *z*=*ω*+i*t* allows a direct calculation to show that, when evaluated with the Gaussian window *g*(*t*) defined in (2.3), the STFT (2.2) can be rewritten as
2.14
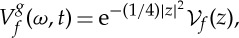
where
2.15
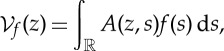
with the kernel
2.16
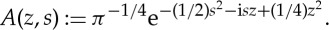
This corresponds to the Bargmann factorization of the STFT, with (2.15) the Bargmann transform of *f* [17], which happens to be an entire function of order 2. As a consequence, the Weierstrass–Hadamard theorem [18] guarantees that 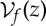 is completely characterized by the distribution of its zeros, according to a factorization whose most general form reads
2.17
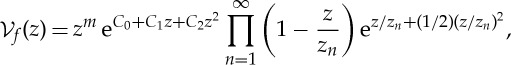
where *C*_0_, *C*_1_ and *C*_2_ are normalization, translation/rotation and squeezing factors respectively, and *m* is the possible multiplicity of a zero at the origin of the plane [19]. Because the zeros *z*_*n*_ of 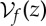 also correspond to the zeros of the STFT, the latter is fully characterized by its zeros.

## 3. Time–frequency segmentation for mode separation

Here, we investigate two different techniques to compute the basin of attraction (or domain) associated with one mode and then base our mode reconstruction on this approach. The first technique involves the use of the direction of RV to define the ridges and then the properties of the RV in the vicinity of the detected ridges, whereas the second is based on a study of the zeros of the STFT.

### (a) Determination of basins of attraction based on ridges and reassignment vectors

There exist many different ways of computing the ridges associated with the TF representation given by the STFT [14,15]. The ridge detector proposed here is based on the properties of RV. In this paper, we note that *ridge points* (RPs) are associated with a sharp variation in the direction of the RV. These can be computed by considering the projection of the RV onto a predefined direction associated with the angle *π*/2+*γ* [9] and then the zeros of the projected vector, i.e.
3.1
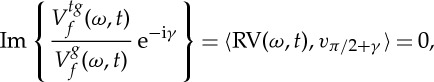
where *v*_*λ*_ is the unit vector in the direction *λ*. The RPs thus correspond to points where the inner product of the RV with unit vector in the direction *π*/2+*γ* changes sign.

Because 
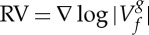
 (see (2.13)), 〈*RV* (*ω*,*t*),*v*_*π*/2+*γ*_〉 can be viewed as the directional gradient in the direction *π*/2+*γ*. Because, in (3.1), the direction *γ* is fixed *a priori*, the method is not adaptive. To improve the adaptability of the method, a variant was proposed in [10] which consisted of modifying the definition of RPs as follows:
3.2
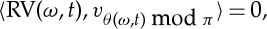
with RV(*ω*,*t*)=*r*(*ω*,*t*) e^i*θ*(*ω*,*t*)^ and where *θ*(*ω*,*t*) mod *π* belongs to [0,*π*[. It is worth noting here that when RV belongs to [0,*π*[ (resp.]−*π*,0]), 〈RV(*ω*,*t*),*v*_*θ*(*ω*,*t*) mod *π*_〉 equals 1 (resp. −1).

**Figure 1.**
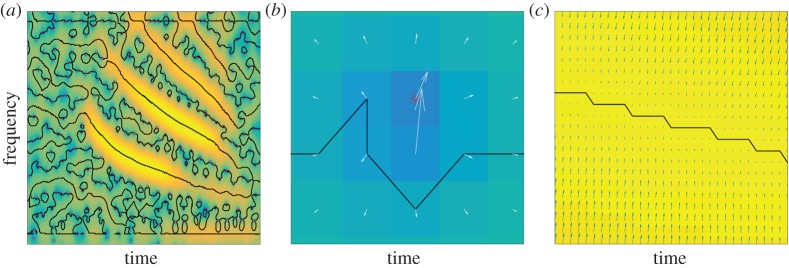
(*a*) Signal spectrogram of a bat signal with the zeros and the ridges superimposed. (*b*) RV close to a zero of the spectrogram (red asterisk); white arrows represent the RV and the nearby contour is plotted in black.(*c*) RV close to a ridge of the spectrogram; blue arrows represent the RV and the ridge is plotted in black. (Online version in colour.)

The underlying rationale for this construction is that, on each side of a ridge, the RV points in opposite directions. The behaviour of RV in the vicinity of a ridge associated with TF representation of figure 1*a* is displayed in figure 1*c*. So from one perspective, the ridge can be viewed as an attractor for the reassignment vector field. The RPs defined in (3.2) are not only associated with ridges but form more general structures in the TF plane, which we call *contours*. These contours do not branch, but terminate on the borders of the TF axes, or form closed loops as discussed in [9]. In addition, the phase along a contour varies smoothly until it passes through a singularity in the zeros of the STFT. The method then segments the contours whenever they cross the zeros of the STFT. This raises the question: why do contours necessarily pass through zeros of the STFT? The RV as stated in expression (2.13) is oriented, in the vicinity of the zeros of the spectrogram (of a bat signal) displayed in figure 1*a*), as shown in figure 1*b*. Note that this behaviour for RV in the vicinity of the zeros of the spectrogram is independent of the signal studied. Consequently, the zeros can be viewed as repellers for the reassignment vector field. Then, owing to the ‘mod *π*’ term in the definition (3.2), 〈RV(*ω*,*t*),*v*_*θ*(*ω*,*t*) mod *π*_〉 is positive above a zero and negative otherwise. Thus, the contour is horizontal in the vicinity of these points. Note that, owing to TF discretization, zeros of the spectrogram cannot be exactly determined; therefore a contour, in practice, passes in the vicinity of the discrete minimum but not necessarily exactly through it.

Another interesting aspect of the proposed method lies in the fact that the contour does not necessarily last for the whole time span of the signal: if the energy becomes locally too small the ridge ends on the nearby zeros of the STFT. This last point is very important because it indicates that a mode dies when it connects with a zero. Usually, the presence or otherwise of a mode is assessed in terms of the statistics of the spectrogram of the noise. The basic idea being that, in order to qualify as a mode, the points should not obey this statistic, and an ad hoc machinery is required to connect such points [20]. In our framework, it is not necessary to perform any statistical analysis to characterize the birth and death of a mode, because our study is based on the locations of the zeros. An illustration of this phenomenon is given in figure 1*a*, where the contours and the zeros are superimposed on the spectrogram of a noisy bat signal.

Finally, to build the basin of attraction knowing the location of a contour, we consider the most frequently occurring contour in the vicinity of 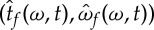. Each point (*ω*,*t*) is then given the index of the ridge it is attached to, and the set of points attached to ridge *i* is denoted by 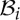. Note here that the notion of basin of attraction is one way to recover the information associated with one mode, and there exist several other ways of gathering this information, as will be discussed later.

### (b) Determination of mode domains based on Delaunay triangulation upon zeros

**Figure 2.**
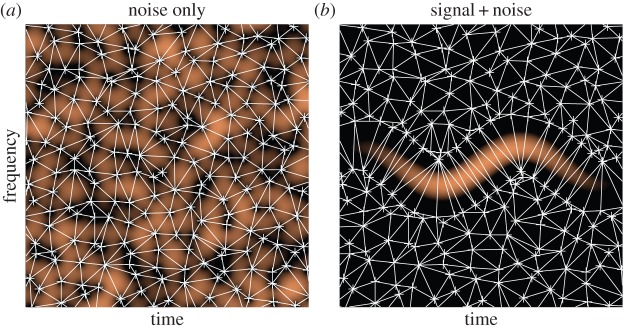
(*a*) Delaunay triangulation based on the zeros of the spectrogram for a noise signal. (*b*) Delaunay triangulation based on the zeros of the spectrogram of a mode superimposed onto the noise of the left diagram. (Online version in colour.)

The second approach to determining a TF domain attached to a given mode arises from noting that the zeros of the STFT completely characterize it. Consequently, it is natural to consider these as a two-dimensional point process in the TF plane, with properties associated with the specific nature of the analysed signal. So, initially, we consider a simplified, geometrical description of the TF structure of a signal by considering the diagrams connecting STFT zeros, i.e. the so-called *stellar representation* used in quantum mechanics [19,21] (see also [22] for a related TF perspective). The simplest approach is to use Delaunay triangulation [23]. When these diagrams are reviewed for the case of white Gaussian noise using the Delaunay triangulation approach, a homogeneous two-dimensional random field defined by the STFT zeros results and the distribution of the zeros is itself homogeneous over the plane. It is expected that this will be no longer the case for a signal with a coherent TF structure, e.g. with superimposed frequency modulation. Figure 2 illustrates that this is exactly what happens: when an AM–FM chirp is added to the noise of the left diagram, the noise-only regions remain unaffected, whereas the signal domain is characterized not only by large spectrogram values, but also by Delaunay triangles with longer edges than in noise-only regions. Note that the addition of coherent signal structure to incoherent noise does not change the total number of zeros in the plane, but simply modifies their distribution, so that they tend to align along the border of mode domains which tend to identify with the previously defined basins of attraction.

Theoretical considerations and the evidence displayed in figure 2 suggest that signal domains could be identified by considering Delaunay triangles that depart from the expected behaviour associated with noise. In [11], it was shown that the distribution of edge lengths of Delaunay triangles constructed using STFT zeros for the case of white Gaussian noise is essentially bounded above by a maximum value 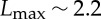 (in the system of normalized units corresponding to the window (2.3)), with a very low probability of exceeding 2 (referring to |*e*_*mn*_| as the distance between any two zeros *z*_*m*_ and *z*_*n*_ in a Delaunay triangle, a numerical evaluation evidenced that 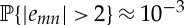). Selecting Delaunay triangles on the basis of thresholding their maximum edge length is therefore a simple way of identifying elementary local domains whose concatenation defines global mode domains 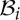 via supports delineated by zeros. It is important to note that the choice of the circular window (2.3) is mandatory to ensure a rotation-invariant analysis. As for signal components, this guarantees to consider in the same way any time–frequency trajectory that is (locally) linear, whatever its orientation: ‘horizontal’ for tones, ‘vertical’ for impulses or ‘oblique’ for chirps. As for the noise, the time–frequency isotropy of the circular window permits setting one unique threshold to determine outlier edges in triangles.

### (c) Mode reconstruction

Whatever the method (ridges or zeros), having computed the different basins 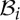 corresponding to the different modes of the signal, the reconstruction of the mode associated with basin *i* is then performed by means of the classical inversion formula
3.3
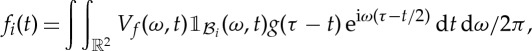
where 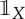 is the indicator function of *X*.

### (d) Comparison of the ridge and zeros based method for the computation of basins of attraction and mode domains

**Figure 3.**
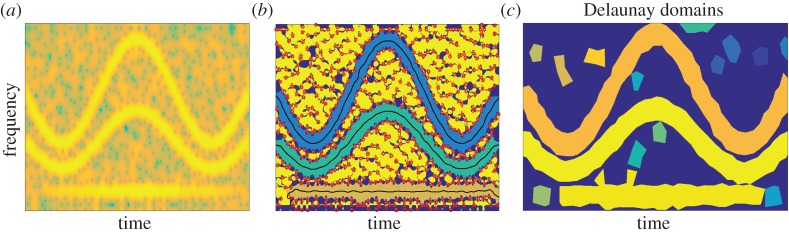
(*a*) Spectrogram of synthetic three-mode signal with additive Gaussian noise (*SNR*=10 dB). (*b*) Ridges and basins of attraction computed as explained in §3a. (*c*) Mode domains computed using Delaunay triangulation as explained in §3b. (Online version in colour.)

We now compare the techniques based on the computation of the basins of attraction using ridge location and RV direction to that based on the mode domains resulting from the Delaunay triangulation of the zeros of the spectrogram. To illustrate the different behaviours, we first consider a synthetic noisy three-mode signal, each mode having sinusoidal phase. We display in figure 3*a* the spectrogram of the signal along with the ridges and basins of attraction corresponding to each mode on figure 3*b* and finally the mode domains based on Delaunay triangulation for the same noisy signal on figure 3*c*. We note that as expected the TF domains associated with the three modes are correctly estimated, allowing a good separation of the modes even when the noise level is relatively high (10 dB in this example).

## 4. Numerical validation

Here, we investigate the properties of the proposed techniques for mode identification and reconstruction for signal denoising as well as illustrating a sound processing application [24]. We have selected two kinds of signal examples, not only for their intrinsic TF properties that fit the AM–FM multicomponent model well, cf. equation (2.4), but also for their ability to cover a large class of musical sounds [25] (and more generally, the class of signals produced by vibrating structures [26]). These are namely the damped sinusoid (with a smooth or sharp attack) related to percussive signals, i.e. the impulse response of a resonant structure, and an excerpt of a cello sound, a typical case of a sustained harmonic signal [27].

### (a) Denoising of a damped tone

**Figure 4.**
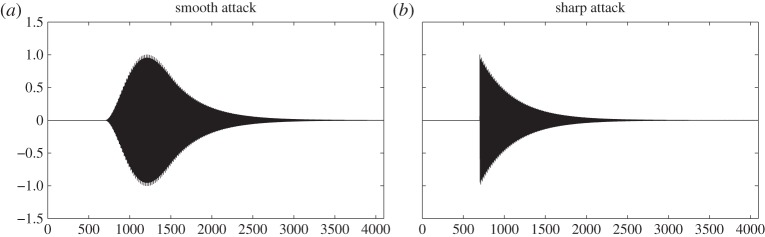
Damped tone with (*a*) a smooth attack (raised cosine) and (*b*) a sharp attack (step function).

We first study the denoising performance of the proposed algorithms, based on determining the basins of attraction and mode domains for reconstruction, on a damped tone that is a simple and widely used model, e.g. in audio [28]. Two cases are considered, with either a smooth attack (raised cosine) or a sharp one (step function); see respectively figure 4*a*,*b*.

**Figure 5.**
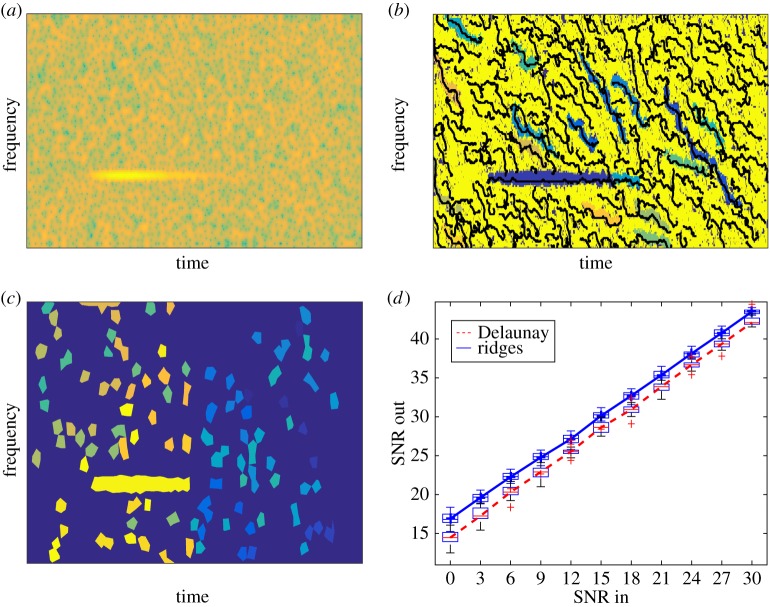
(*a*) Spectrogram of a noisy damped tone with a smooth attack (SNR= 0 dB). (*b*) Main ridges and basins of attraction derived from the RV. (*c*) Mode domains derived from Delaunay triangulation. (*d*) Denoising performance of the mode reconstruction. (Online version in colour.)

In the first case, we display the spectrogram of such a signal contaminated by additive white Gaussian noise on figure 5*a*, the basins of attraction with respect to the energy of the STFT computed on their associated ridges in figure 5*b* and the Delaunay domains in figure 5*c*. Finally, figure 5*d* presents the denoising performance for both methods. The output signal-to-noise ratio (SNR) is computed by comparing the original signal to the mode reconstructed using the most energetic basin of attraction for the method based on RV (3 in the case of Delaunay triangulation). For the Delaunay triangulation method, the choice of considering the three most energetic domains accounts for a possible splitting of the main domain due to the non-detection of one or two triangles. In that example, both methods behave in a very similar fashion.

**Figure 6.**
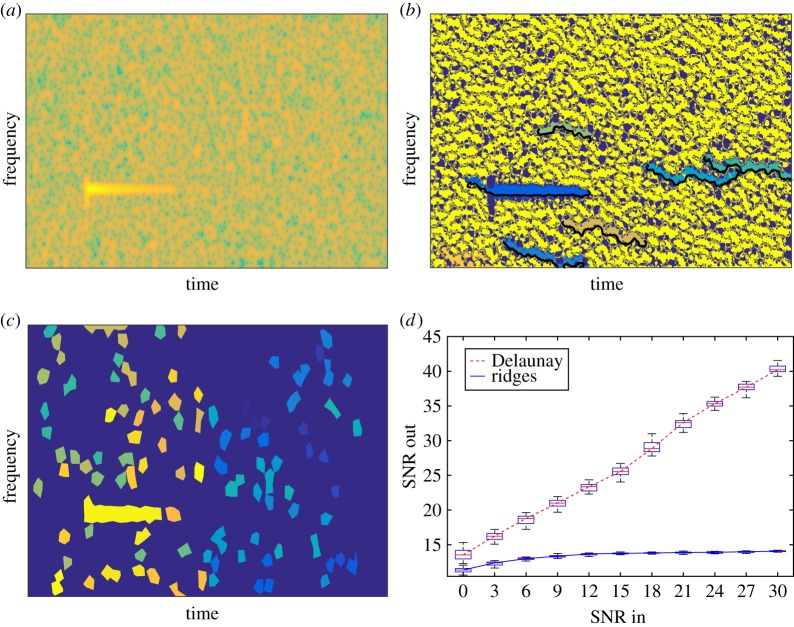
(*a*) Spectrogram of a noisy damped tone with a sharp attack (SNR= 0 dB). (*b*) Main ridges and basins of attraction derived from the RV. (*c*) Mode domains derived from Delaunay triangulation. (*d*) Denoising performance of the mode reconstruction methods. (Online version in colour.)

A similar analysis is provided for the case of the sharp attack (figure 6). The damped tone with a sharp attack exhibits a TF representation made of a horizontal and a vertical part, the latter corresponding to the attack. What is interesting about this signal is that it no longer satisfies (2.4), because the amplitude of the mode is no longer differentiable. The behaviours of the two methods on this type of signal are significantly different. While the method based on the Delaunay triangulation manages to capture the horizontal and the vertical parts of the mode, the method based on RV never acquires the attack (even at low noise levels). This results in an output SNR which is much worse for the latter compared with the former method. Note that the vertical TF structure could be detected using the method based on RV by changing *θ*(*ω*,*t*) mod *π* in (3.2) into *θ*(*ω*,*t*)+*π*/2 mod *π*−*π*/2, but then two different analyses would be required to properly analyse such a signal. An alternative method, connected with the RV, will be investigated in the near future; this will be based on an analysis using bidimensional ridge detection [29].

### (b) Analysis of a cello sound

**Figure 7.**
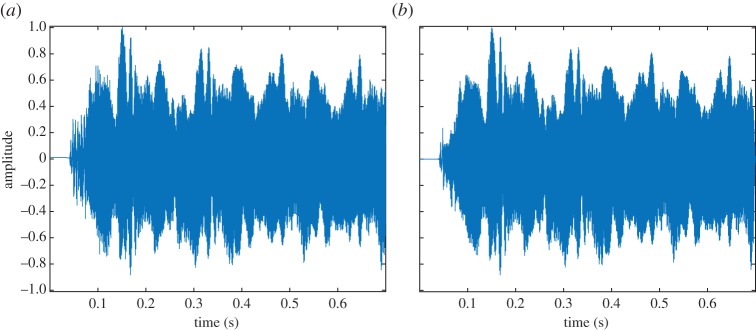
(*a*) Cello sound, original signal; and (*b*) cello sound, reconstructed signal after mode domain extraction derived from Delaunay triangulation. (Online version in colour.)

**Figure 8.**
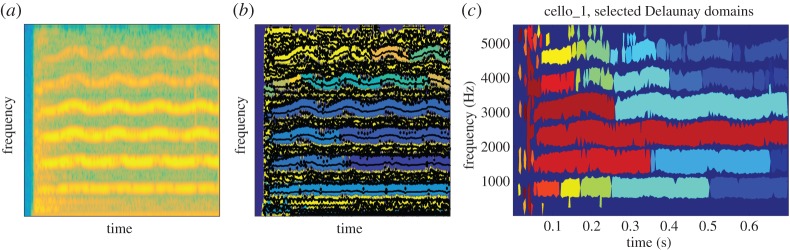
(*a*) Spectrogram of a cello sound. (*b*) Main ridges and basins of attraction derived from the RV. (*c*) Mode domains derived from Delaunay triangulation. (Online version in colour.)

We now apply the techniques proposed in this paper to the problem of identification and extraction of the harmonic structure of the first part of a cello note, a G5 (a frequency of 776 Hz). The temporal structure of the example (sampled at 11 025 Hz and lasting for 0.7 s) is relatively complex and shows a fast attack followed by some AM–FM modulations (cf. figure 7*a*). Its spectrograms are easier to decipher as it clearly exhibits the first six partials that lie within the available frequency range [0,55 012.5] Hz (figure 8*a*). Note the vibrato effect, which appears as a pseudo-sinusoidal modulation of the fundamental frequency and results in a frequency deviation that increases proportionally to the harmonic rank. Also note the presence of noise during the note attack around 0.05 s; this is produced by the bow rubbing the string while the oscillatory motion takes place [27].

For the RV-based analysis (figure 8*b*), we verify once again that the basins of attraction are easily determined for high-amplitude modes; this is, indeed, the case for the first (i.e. the fundamental) and the fourth. For the other modes, spurious zeros in the spectrogram might cause the splitting of the basin associated with a single mode into several smaller basins. Note that, while the algorithm detects the modes, reconstructing them based on their basins of attraction would require some post-processing steps in order to group them in some relevant way.

In the case of the mode domain extraction based on the Delaunay triangulation, each of the six harmonic partials is clearly identified by corresponding domains that track frequency modulations well (figure 8*c*). The highest-amplitude partial is entirely represented by only one domain, while each of the others is represented by a few subdomains, usually due to a missing triangle that breaks the continuity of the whole. As in the case of the RV-based method, a post-processing step would help merging subdomains to reconstitute the domain that represents a whole partial. In addition, the Delaunay-based method confirms its ability to detect and render transients. Indeed, note the vertical structure centred around the attack time (0.05 s, figure 8*c*).

Finally, an experiment was performed to reconstruct the harmonic and attack structure of the cello sound by applying the reconstruction technique, i.e. equation (3.3), with masking domains identified via the Delaunay technique. One can see the temporal representation of the reconstructed signal in figure 7*b*, which looks very similar to the original excerpt (cf. figure 7*a*). An auditory comparison confirms how close the two signals are to each other (cf. electronic supplementary material).

In terms of post-processing, for both the RV and Delaunay-based methods, note that when the modes are harmonically related, one can benefit from the fact that the instantaneous frequencies of harmonic components are multiples of the fundamental frequency to gather together basins of attraction/mode domains associated with a given mode. Note also that this pertains to the problem of reconstructing trajectories of partials in the context of additive synthesis [30]. This is a topic that we will seek to address in the near future. A fruitful application for this harmonic identification/extraction is the separation of the noisy part from the pseudo-periodic part of any given sound. This is a key step in preserving the perceptual naturalness and in achieving high-quality results in advanced audio processing techniques such as time-scaling, transposition or sound morphing [31]. Indeed, one has to process differently the pseudo-deterministic part from the noisy part (e.g. for time-scaling and transposing sounds, the noisy attack part has to remain unchanged).

## 5. Conclusion

In this paper, we have presented two different STFT-based mode decompositions. The first estimates the *ridges*, then computes the basins associated with the modes by making use of the reassignment vector. The second is more novel, because it uses only the location of the STFT’s zeros to build these domains. Both methods provide a segmentation of the TF plane into meaningful components, and a reconstruction of these modes in a fully adaptive way. The experiments presented here show that both methods can decompose multicomponent signals with high accuracy, even when the signals are contaminated by noise. The comparisons also show that the ridge-based reconstruction can be more precise, whereas the Delaunay-based segmentation allows for more complex modes (e.g. containing sharp attacks or impulses). To conclude, we note that such TF adaptive decompositions can be easily applied to time-scale representations (e.g. continuous wavelet transform), which is more appropriate for some particular modulations and intermode separation. Similarly, it should be possible to adapt such decompositions to non-harmonic signals. This will be the subject of further study, which will also focus on real-life applications, e.g. in automatic music signal analysis.

## Supplementary Material

ESM_paper_0507.pdf

## Supplementary Material

signals.zip

## Supplementary Material

mcs_ridge_decomposition.zip

## Supplementary Material

spz_Delauny_Package.zip
