# Supplementary material for: Adaptive multimode signal reconstruction from time–frequency representations
Source: Philos Trans A Math Phys Eng Sci. 2016 Apr 13;374(2065):20150205. doi: 10.1098/rsta.2015.0205 (PMC4792411; doi:10.1098/rsta.2015.0205)
Supplement: ESM_paper_0507.pdf [file rsta20150205supp1.pdf]

PHILOSOPHICAL TRANSACTIONS A

rsta.royalsocietypublishing.org

Research

Article submitted to journal

Subject Areas:
Applied Mathematics, Signal Processing

Keywords:
Time-Frequency, AM-FM signals, Reassignment, Multimode signal reconstruction

Author for correspondence:
Stephen McLaughlin
e-mail: s.mclaughlin@hw.ac.uk

Electronic Supplementary Material for Adaptive multimode signal reconstruction from time-frequency representations

Stephen McLaughlin[1], Sylvain Meignen[2], Thomas Oberlin[3], Philippe Depalle[4] and Patrick Flandrin[5]

[1]School of Engineering and Physical Sciences, Heriot-Watt University, Edinburgh Scotland, UK
[2] LJK, University of Grenoble, France
[3] IRIT,University of Toulouse, France
[4] SPCL,McGill University, Montréal, Canada
[5] SISYPHE, ENS Lyon, France

THE ROYAL SOCIETY PUBLISHING

© The Authors. Published by the Royal Society under the terms of the Creative Commons Attribution License http://creativecommons.org/licenses/by/4.0/, which permits unrestricted use, provided the original author and source are credited.

This document summarises the Electronic Supplementary Material supplied to support our paper on Adaptive multimode signal reconstruction from time-frequency representations. The material consists of the signals used to illustrate our method and all associated code.

1. Signals used in analysis

- Bat echolocation signal
- Cello sound

2. MATLAB code and associated brief description

Note that the code is made available to assist readers in understanding our methods, it comes with no warranties and as with all code applying it is no substitute for understanding what it aims to do, so *caveat emptor*.

(a) Delauny - General Description

This piece of software implements the ideas proposed by P. Flandrin, described in the paper, for signal analysis and filtering, based on STFT zero detection, and Delaunay triangulation.

The main script is *spz_delaunay_example_modular*, which runs the whole process of STFT zero detection, Delaunay triangulation, mode domain construction, mode selection, and mode reconstruction on a few selected signal examples. *spz_delaunay_example_modular* is, by default, configured to automatically run on an exponentially damped sinusoids with a smooth attack (namely: Damped Tone, - Cosine attack). *spz_delaunay_example_modular* displays figures of the relevant elements: original signal, spectrogram, all resulting triangles, selected triangles (the ones that correspond to modes), extracted mode domains, energy of the domains, selected mode domains, temporal representation of the reconstructed signal from selected domains, and signal difference between original signal and reconstructed one. Other examples can be run by commenting the Damped Tone - Cosine attack section, and uncommenting the section selectively. Users can also apply the process on their own signal by mimicking existing examples.

A few practical details: *pz_delaunay_example_modular* has to be launched from within the directory spz_Delaunay_Package unless *spz_Delaunay_Package* is added to the MATLAB path. *spz_delaunay_example_modular* saves files in a directory called Tmp, which has to be created before the first run (*spz_delaunay_example_modular* will warn you if this was not done). *spz_delaunay_example_modular* allows you to run subparts of the process only by setting appropriate flags at true or false (please see the flag section in the script - lines 50 to 85- for corresponding actions.)

Required software package: The Time-Frequency Toolbox (TFTB) by Francois Auger, Olivier Lemoine, Paulo Goncalves and Patrick Flandrin; available at http://tftb.nongnu.org

(b) Ridge based code description

The Matlab package *mcs_ridge_decomposition.zip* provides the Matlab implementation of the ridge-based decomposition method described in [1,3].

The main Matlab function is *get_contour_basins.m*. The package also contains three scripts (*demo_figure3.m, demo_figures5and6.m, demo_figure8.m*) that draw the figures of paper [1].

It uses some functions of the TFTB toolbox [2], which were included to make the use easier (functions *amgauss, dwindow, roundgauss, sigmerge, tfrstft, tfrrsp, tfristft*)

Three small datasets are included: *Damped_Tone_Noise.mat, Cello_1.wav, and mcs_3modes.mat* (see [1] for a detailed description)

Note that this code is provided in order to make the experiments of [1] easily reproductible. It is not a general toolbox for time-frequency analysis and mode reconstruction.

3. References

[1] Adaptive multimode signal reconstruction from time-frequency representations, by Sylvain Meignen, Thomas Oberlin, Philippe Depalle, Patrick Flandrin, and Stephen McLaughlin, submitted.

[2] The Time-Frequency Toolbox (TFTB), by Francois Auger, Olivier Lemoine, Paulo Goncalves and Patrick Flandrin, available at http://tftb.nongnu.org/

[3] Time-frequency ridge analysis based on reassignment vector, by Sylvain Meignen, Tim Gardner and Thomas Oberlin, in Proceedings of the 23st European Signal Processing Conference (EUSIPCO-15), 2015.
